# Micro- and Nanoplastics as Disruptors of Digestive and Hepatopancreatic Homeostasis: Insights into the Plastic-Gut-Liver Axis

**DOI:** 10.3390/ijms27073272

**Published:** 2026-04-04

**Authors:** Nicoletta Capuano, Martina Lombardi, Noemi Cafà, Marianna Marino, Flora Salzano, Federica Scalia, Raffaele Marfella, Giovanni Villone, Francesco Cappello, Marta Anna Szychlinska, Gianluigi Franci, Antonietta Santoro, Luca Rinaldi

**Affiliations:** 1 Department of Medicine, Surgery and Dentistry “Scuola Medica Salernitana”, Università di Salerno, 84081 Baronissi, Italy; niccapuano@unisa.it (N.C.); marlombardi@unisa.it (M.L.); ncafa@unisa.it (N.C.); mariannamarino6@gmail.com (M.M.); flsalzano@unisa.it (F.S.); gfranci@unisa.it (G.F.); 2 Department of Experimental Medicine, University of Campania “Luigi Vanvitelli”, 80138 Naples, Italy; 3 U.O.S. Microbiology and Virology, A.O.U. “San Giovanni di Dio e Ruggi d’Aragona”, 84131 Salerno, Italy; 4 Department of Medicine and Surgery, University Kore of Enna, 94100 Enna, Italy; federica.scalia@unikore.it; 5 Department of Advanced Medical and Surgical Sciences, University of Campania “Luigi Vanvitelli”, 80138 Naples, Italy; raffaele.marfella@unicampania.it; 6 Department of Medicine and Health Sciences “Vincenzo Tiberio”, University of Molise, 86100 Campobasso, Italy; giovanni.villone@unimol.it (G.V.); luca.rinaldi@unimol.it (L.R.); 7 Department of Biomedicine, Neuroscience and Advanced Diagnostics (BIND), University of Palermo, 90127 Palermo, Italy; francesco.cappello@unipa.it; 8 Department of Precision Medicine in Medical, Surgical and Critical Care (MEPRECC), University of Palermo, Via del Vespro N. 129, 90127 Palermo, Italy

**Keywords:** micro- and nanoplastics, gut–liver axis, pancreatic dysfunction, gut microbiota, dysbiosis, environmental exposome

## Abstract

Micro- and nanoplastics (MPs/NPs) have emerged as pervasive environmental contaminants with increasing implications for human health, particularly within the digestive system. This review critically examines the role of MPs/NPs as disruptors of gastrointestinal and liver homeostasis through the lens of the plastic–gut–liver axis. We synthesize current evidence on primary exposure routes—including ingestion, inhalation, dermal contact, and transplacental transfer—and highlight their intestinal uptake, systemic dissemination, and tissue accumulation. Mechanistically, MPs/NPs compromise intestinal barrier integrity, promote oxidative stress, and induce microbiota dysbiosis, facilitating the translocation of microbial-derived signals to the liver via the portal circulation. This process triggers inflammatory signaling cascades, metabolic reprogramming, and immune dysregulation, contributing to hepatic steatosis, insulin resistance, and potential carcinogenic processes. Emerging evidence also implicates pancreatic dysfunction and β-cell stress within a broader gut–liver axis context. We further discuss the systemic propagation of MPs/NPs-induced dysbiosis along multi-organ axes, including gut–lung and gut–brain interactions. Despite robust preclinical data, human evidence remains limited due to methodological heterogeneity and the lack of standardized biomarkers. This review underscores critical knowledge gaps and emphasizes the need for integrative, translational approaches to clarify long-term health risks and inform regulatory strategies within the environmental exposome framework.

## 1. Introduction

Micro- (MPs, <5 mm) and nanoplastics (NPs, <100 nm) have emerged as ubiquitous environmental contaminants with growing relevance for human health. Their widespread distribution across aquatic, terrestrial, and atmospheric ecosystems has resulted in continuous and unavoidable human exposure, primarily through ingestion [1,2]. Despite increasing knowledge, the implications of chronic low-dose exposure to MPs/NPs for human digestive health remain insufficiently characterized. The gastrointestinal (GI) tract represents the principal interface between MPs/NPs and the human body. Beyond acting as a passive site of exposure, the gut plays an active role in mediating particle uptake, immune sensing, and systemic dissemination [3]. Experimental evidence indicates that MPs/NPs disrupt intestinal barrier integrity, alter mucus composition, and induce oxidative stress and local inflammation. These effects are further exacerbated by alterations of the gut microbiota, leading to dysbiosis, increased intestinal permeability, and enhanced translocation of microbial-derived products [4,5].

Through the gut–liver axis, MPs/NPs-induced intestinal dysfunction spreads inflammatory and metabolic cues to downstream organs, particularly the liver, which is highly susceptible to portal-derived insults. Preclinical studies consistently demonstrate that MPs/NPs accumulate in hepatobiliary tissues, where they interfere with lipid and glucose metabolism, mitochondrial homeostasis, and immune regulation in both aquatic organisms and mammalian models [6,7,8,9]. The activation of MPs/NPs-induced stress-responsive signaling pathways commonly associated with apoptosis, and pyroptosis, has been reported in response to MPs/NPs exposure. However, studies investigating their role in chronic liver disease, metabolic dysfunction, and hepatocarcinogenesis are still in their infancy [10,11].

Despite the expanding body of mechanistic evidence, significant gaps persist. Human epidemiological data are scarce, standardized exposure metrics are lacking, and clinically validated biomarkers of MPs/NPs exposure and tissue damage have yet to be established. Moreover, the potential bidirectional transport of MPs/NPs—from the gut to the liver via the portal circulation, and from the liver back to the intestine through hepatobiliary and pancreatic tracts—has not yet been thoroughly investigated, although this process may further amplify their biological effects [12,13]. In addition, the long latency periods characteristic of chronic digestive and hepatopancreatic diseases complicate causal inference, underscoring the need for integrative experimental and translational approaches.

In this review, we provide a comprehensive synthesis of current knowledge on the impact of MPs/NPs on the GI tract and gut–liver axis, with a particular focus on molecular, immunological, and microbiome-mediated mechanisms. We examine routes of exposure, tissue accumulation, digestive system-specific effects, and inter-organ communication axes, highlighting emerging evidence that positions MPs/NPs as relevant components of the environmental exposome able to affect gut, liver and pancreas homeostasis. However, assessing the biological impact of MPs/NPs in humans is inherently challenging, as real-world exposure occurs in the context of complex mixtures of environmental contaminants, including both plastic particles and non-plastic pollutants. This co-exposure complicates the attribution of specific biological effects to MPs/NPs. By integrating current preclinical evidence with emerging human data, this review aims to highlight key knowledge gaps and priorities for future translational research.

## 2. MPs/NPs Primary Exposure Routes and Biological Impacts

The ubiquitous presence of MPs/NPs across all environments poses a significant challenge to both environmental and human health, further magnified by their capacity to act as carriers for harmful substances. Extensive research has focused on the mechanisms of human exposure, primarily identifying three main routes: ingestion, inhalation, and dermal contact [14,15]. Emerging evidence requires the inclusion of transplacental transfer as a critical fourth route. This newly identified exposure route, combined with the fragile nature of fetal development, highlights a significant and under-studied threat to human health [16,17,18].

### 2.1. Ingestion

Ingestion is recognized as the primary route for MPs/NPs entry into the human body, occurring directly via contaminated water and indirectly through the food chain [15]. The detection of these particles in a wide variety of foods raises significant concern regarding their ability to traverse the GI barrier and enter systemic circulation [19,20]. In this context, several mechanisms have been proposed to influence MPs/NPs GI absorption such as their ability to interact with molecules and variable physicochemical features that can influence their translocation potential. For instance, during digestion, MPs/NPs interact with various biomolecules (e.g., proteins, lipids, carbohydrates), leading to the formation of the so-called “protein corona”. This structure alters the particles’ surface chemistry, charge and hydrophobicity, essentially providing a “biological disguise” that facilitates their translocation into the circulatory and lymphatic systems, potentially leading to accumulation in distant tissues [21]. Moreover, intrinsic particle characteristics such as surface charge and size profoundly influence translocation capacity. Studies on intestinal co-cultures of Caco-2 and HT29-MTX cells indicate that positively charged NPs exhibit a fourfold greater translocation ability than neutral ones, while negatively charged NPs demonstrate an increase in uptake potential up to an 80-fold [22]. Regarding size, it has been shown that decreasing particle size correlates with increased uptake. Particles larger than 20 µm generally remain confined to the GI tract and are excreted. Smaller MPs can be absorbed by the gut and reach the circulatory system, while NPs, particularly those less than 100 nm, show a higher propensity to cross the GI barrier and be internalized by cells [23].

Despite the growing body of evidence describing ingestion-related exposure, several limitations remain. For instance, in aquatic ecosystems, MPs/NPs have been shown to act as vectors for toxic chemical compounds, including dichlorodiphenyltrichloroethane (DDT), polybrominated diphenyl ethers (PBDEs), and heavy metals such as Pb, Zn, and Cd [24,25]. This carrier function complicates the accurate determination of actual exposure levels to MPs/NPs and hampers the assessment of their individual, synergistic, or combined effects on human health when co-occurring with other toxic substances.

### 2.2. Inhalation

Inhalation is the second major entry route, largely due to the release of airborne particles resulting from the degradation and wear of plastic materials. MPs/NPs have been identified in various biological samples, including sputum and bronchoalveolar lavage fluid (BALF) [26]. In this context, the lungs serve as a critical human body entry point. Once inhaled, MPs/NPs can be internalized by human alveolar epithelial cells, activating inflammatory gene transcription. Indeed, polystyrene (PS) NPs induced significant upregulation of pro-inflammatory cytokines such as IL-8, NF-κB, and TNF-α, as well as pro-apoptotic proteins such as DR5, caspase-3, 8 and 9, and cytochrome c in human A549 cells, suggesting that PS particles are responsible for respiratory injury [27]. Accordingly, the exposure of mice to polymethyl methacrylate (PMMA) by intratracheal intubation revealed increased cell count and elevated inflammatory cytokines in serum and BALF associated with lung tissue alterations [28]. This scenario is further complicated by the possibility that NPs could cross the alveolar–capillary barrier and enter the systemic circulation, enabling hematogenous dissemination to multiple organs. The translocation process can be influenced not only by physicochemical properties—such as particle size, surface charge, and hydrophilicity—but also by increased epithelial and endothelial permeability, as occurs during preexisting inflammatory or immune activation.

In occupational settings, workers face significantly higher concentrations of MPs/NPs than the general population, primarily through inhalation [29,30]. Chronic exposure, occurring in the presence of co-exposures to other toxic agents and pre-existing pathologic conditions, may amplify inflammatory responses and increase the risk of respiratory disease. Notably, inhaled particles cleared via the mucociliary escalator can be swallowed, contributing to secondary gastrointestinal exposure [31].

Within the framework of the gut–liver axis, this dual exposure route may facilitate the transfer of inhaled MPs/NPs from the respiratory system to the GI, potentially promoting intestinal barrier dysfunction and the subsequent propagation of inflammatory signals to the liver. Despite these concerns, the lack of specific occupational exposure limits and the limited understanding of MPs/NPs bioavailability and toxicity highlight a critical regulatory gap.

### 2.3. Dermal Contact

Dermal absorption is generally considered a less efficient route but remains relevant, especially in cases of skin barrier disruption [32]. Introduction occurs via cosmetics, personal care products, pharmaceutical products, and contact with contaminated water. Due to their hydrophobic nature, probable absorption pathways include sweat glands, hair follicles, wounds, abrasions, and skin lesions [33]. The wear of surgical or dental implants also constitutes a slow but continuous release source, leading to subsequent particle migration into circulation [34]. In occupational and clinical contexts, iatrogenic exposure represents a more significant source of systemic MPs/NPs. Disposable medical devices (e.g., syringes, catheters, infusion sets)—made from polymers like polypropylene (PP), polyvinyl chloride (PVC), and polyethylene (PE)—also represent a significant source of exposure [35,36]. Casella et al. detected MPs (fragments and fibers, >60% and <100 µm) in nearly all intravenous infusion fluids, identifying concentrations >200 MPs/L as indicative of high clinical risk [37]. In addition, Chen et al. demonstrated that normal blood collection procedures release numerous polymer types (e.g., polyurethane, PU; ethylene vinyl acetate, EVA; polyamide, PA; PVC; polyethylene terephthalate, PET), with higher temperatures (37 °C) promoting the release of smaller particles (10−30 µm) [38]. Notably, recent observations in healthy individuals link circulating MPs/NPs with increased use of plastic food containers, correlating with alterations in coagulation parameters such as elevated C-reactive protein, prolonged partial prothrombin time (aPTT), and increased fibrinogen levels [39]. Collectively, these findings indicate that, beyond environmental exposure, medical procedures and occupational contact may serve as underrecognized sources of systemic MPs/NPs favoring the establishment and/or exacerbation of inflammatory conditions.

### 2.4. Transplacental Transport

The transplacental pathway is a potential route contributing to fetal and reproductive abnormalities [20]. While ingestion, inhalation, and dermal absorption describe how plastic particles enter the adult body, the maternal-to-fetal route represents a unique “secondary” exposure that bypasses the fetus’s own external barriers. Recent findings indicate that MPs/NPs such as PS NPs can cross the placental barrier in a size-dependent manner [17,40]. As is known, MPs/NPs could serve as vehicles for other pollutants like endocrine disruptors and persistent organic pollutants, promoting exposure of the developing fetus. Because the placenta has limited defenses against foreign particles, this exposure can trigger inflammation and genetic changes, potentially leading to long-term developmental and reproductive disorders [41]. On this hand, intravenous injection of PS NPs into pregnant FVB/N mice at embryonic day 17 showed that PS NPs accumulate in the placenta and are distributed in various fetal organs like the brain, lung and liver [42]. Few studies have investigated human transplacental transport. Aengenheister et al. described an in vitro co-culture model to mimic the human transplacental barrier, which has been used to investigate PS NPs transport. They found that 49 nm PS NPs penetrated the barrier, while 70 nm PS NPs did not [40]. Moreover, a study also reported MPs in human placenta samples, while NPs were not specifically detected due to analytical limitations [43].

Given the relevance of this route, it is crucial to deepen our understanding of how continuous exposure to these materials may affect human development from birth to long-term consequences. Direct confirmation in human populations is still limited, and further studies are needed to clarify the extent, mechanisms, and long-term consequences of prenatal exposure to MPs/NPs.

## 3. MPs/NPs Tissue Accumulation in the Human Body

Post-mortem analyses have shown that NPs (<0.02 µm), including PET, PS, and polyacrylonitrile (PAN), were detected in filtrates, indicating their ability to cross biological barriers and accumulate in multiple organs, with the highest levels detected in the thyroid, followed by the liver and brain [44,45].

Brain tissue analyses have revealed an increased presence of PET-MPs/NPs compared to past data, with concentrations 6–10 times higher in patients diagnosed with dementia than in healthy individuals [46]. A similar difference was observed in the liver, where patients with cirrhosis exhibited plastic fiber levels of 11.9 particles/g, compared to 1.9 particles/g in healthy controls [47,48]. Although the thyroid (40.4 particles/g), followed by the lungs (14 particles/g), remains the primary site of accumulation among the soft tissues, high concentrations of MPs were also detected in the small intestine (9.5 particles/g), large intestine (7.9 particles/g), and tonsils (6 particles/g). The same study suggests that females are more likely to accumulate MPs [46].

However, it is critical to note that quantitative findings are subject to the risk of sample contamination during necropsy. Without the rigorous use of procedural blanks and specialized “clean-room” environments, ambient plastic particles can lead to significant overestimations [49]. Furthermore, the issue of method comparability remains a hurdle. The transition from particle-counting techniques like μ-Raman to mass-based measurements like Pyrolysis gas chromatography mass spectrometry (Py-GC-MS) often yields non-congruent data, complicating the establishment of universal safety limits [50]. In addition, current limitations in NPs detection suggest that the actual burden in these tissues may be significantly higher. Most analytical frameworks are currently limited by around 1 µm resolution, leaving the sub-micron fraction—which possesses higher potential for cellular internalization—largely unquantified. This probably leads to significant underestimation of retained plastic particles [51].

## 4. Mechanisms of MPs/NPs Uptake and Organ-Specific Translocation Effects: Focus on Digestive System

MPs/NPs primarily enter target cells through endocytosis and passive diffusion [52]. The specific endocytic pathway is largely size-dependent. Larger particles ranging from 200 nm up to 3−5 µm are absorbed via micropinocytosis and phagocytosis, while particles smaller than 200 nm typically utilize clathrin-mediated or caveolae-mediated pathways [53]. Efficiency generally decreases as particle size increases and absorption efficiency is then dictated by the MPs/NPs surface properties [22,23].

### 4.1. Intestinal Absorption and Barrier Integrity Alteration

The intestinal mucus layer serves as a critical defensive barrier, restricting MPs/NPs translocation into the bloodstream [5]. Beyond a purely physical function, the mucus operates as part of a dynamic muco–microbiotic layer, in which host-derived mucins and resident microbial communities form an integrated functional interface regulating epithelial protection, immune tolerance, and microbial spatial organization [54]. Its protective role is supported by findings of reduced Reactive Oxygen Species (ROS) production and diminished inflammatory responses when the mucus remains intact. MPs/NPs diffusivity across the mucus layer is primarily governed by their composition, surface charge, and particle size [55]. Biophysically, the relative importance of these factors is dictated by the ratio of the particle diameter to the mucin fiber mesh size (typically 20–200 nm). Steric hindrance is the primary determinant for larger MPs (typically >500 nm), when the diameter exceeds the mesh size, while for NPs smaller than the mesh size (typically <200 nm), surface chemistry and electrostatics become dominant through “filtering” mechanisms: cationic particles are often immobilized by electrostatic attraction to negatively charged sialic acid residues on mucins (muco-adhesion), whereas neutral or polyethylene glycol (PEG)ylated particles may bypass these traps (muco-penetration). Predictive models often utilize the effective diffusion coefficient (Deff) within hydrogel-based matrices to mimic these transport kinetics and account for the viscoelastic properties of the mucus layer [54]. It is important to underline that accurate in vitro study of this barrier remains challenging, necessitating the use of hydrogel-based matrices to mimic its complex structure [56]. Once the gut barrier is breached, MPs/NPs can enter systemic circulation. Their interaction with the mucosal lining causes local lesions, ROS production and a pro-inflammatory status [56]. Critically, MPs/NPs can facilitate bacterial invasion; for example, they can promote the vascular distribution of bacterial metabolites like lipopolysaccharide (LPS), leading to infections and inappropriate immune responses by creating a “plastisphere” habitat, which facilitates microbial growth and acts as a repository for pathogens [57,58]. MPs/NPs act as carriers for toxic substances, released into the intestinal lumen. Compounds such as Bisphenol A (BPA) and phthalates exacerbate intestinal damage and are linked to hepatic and reproductive alterations in animal models [59]. The higher surface-to-volume ratio of PS-NPs compared to PS MPs enhances the adsorption and delivery of pesticides (e.g., FenProPathrin), leading to more severe toxicological effects, as evidenced by alterations in the silkworm (*Bombyx mori*) [60].

### 4.2. Hepatobiliary and Pancreatic Metabolic Disruption

Following absorption across epithelial barriers, MPs/NPs accumulate in the liver, often aided by a “protein corona” of serum albumin that forms in circulation and facilitates translocation into secondary organs [21,61]. Notably, the protein corona is not a static structure but a dynamic and evolving layer whose characteristics continuously change according to the surrounding biological environment. In humans, MPs/NPs reach the liver via the portal vein and interfere with glycolipid metabolism. This is linked to effects on intermediate metabolites, resulting in the overproduction of fatty acids and enhanced lipid metabolism, increasing the risk of metabolic disorders such as type II diabetes, steatosis, and hyperlipidemia [13,62]. Furthermore, it has been evidenced that gilts treated for one month with PET MPs show significant changes in global metabolomic profile of the pancreas, suggesting that PET MPs may contribute to impaired insulin secretion and potentially lead to insulin resistance and pancreatitis [63]. Furthermore, specific exposure to PS NPs has been shown to reduce the expression of Fatty Acid Transport protein 2 (FAT2), thereby suppressing hepatic fatty acid uptake and biosynthesis [64]. While direct evidence of MPs/NPs’ impact on the human pancreas is currently unavailable, insights are drawn from animal models (pigs, mice, and zebrafish). RNA-Seq studies in pigs have revealed a dose-dependent effect of PET MPs on the expression of genes associated with diabetes pathogenesis, alongside increased oxidative and lipotoxic stress and impaired pancreatic exocrine function [65,66]. Mechanistically, emerging evidence suggests that PET particles are transported via serum-derived extracellular vesicles, where they negatively impact systemic body functions by inducing the differential regulation of miRNAs associated with obesity, insulin resistance, diabetes and metabolic syndrome [67]. Elevated levels of cytokines, such as IL-12β, CXCL9, and CXCL10, implicated in pancreatic β-cell damage, have also been observed [68]. This β-cell stress and consequent dysregulation of insulin secretion may further impact hepatic glucose and lipid metabolism, given the central role of insulin in regulating hepatic metabolic homeostasis, thereby functionally linking pancreatic alterations to liver metabolic imbalance within the gut–liver–pancreas axis.

Among the plastic materials in use, PS was associated with hepatotoxicity, contributing to carcinogenicity and hepatocellular carcinoma (HCC) upon prolonged exposure in adult male BALB/c mice. PS MPs (0.5 µm) were found to primarily regulate the cell death-associated genes BAX and CASP8, which are involved in non-alcoholic fatty liver disease (NAFLD) and inflammation. Of note, co-exposure of Cd and MPs exacerbates and accelerates hepatotoxicity, fibrosis and carcinogenesis [11,69]. Chen and coworkers demonstrated that mice fed with 36 and 116 µm diameter PET MPs at a dosage of 100 µg/g of food until 9 weeks exhibited liver cell proliferation, induced steatosis and altered levels of genes involved in uptake, synthesis, and β-oxidation of fatty acids. Accordingly, oral administration of 2 µm PVC MPs (0.5 mg/day) for 60 days to mice also promoted oxidative imbalance, increased inflammatory foci and cytokine expression, thus affecting the liver detoxification response [70]. Furthermore, Golubska et al., demonstrated that exposure of immature piglets to PET-MPs for 4 weeks disrupted systemic homeostasis such as cholesterol metabolism, transferase activity, and oxidation and contributed to liver dysfunction, increasing catalase (CAT) activity and decreasing superoxide dismutase (SOD) and glutathione peroxidase (GPx) activities in the liver [71].

Understanding of MPs/NPs’ effects on the biliary tract is fragmented due to the lack of direct human data, relying heavily on pharmacokinetic animal studies. Murine models have shown that PS MPs/NPs (50 nm size) administered intravenously accumulate in hepatocytes and are subsequently eliminated in the feces or excreted via the biliary route within 24 h [72,73]. In this context, a potential link between MPs and gallstone formation has been preliminarily investigated, highlighting the involvement of cholesterol [73]. A study on murine models fed a high-cholesterol diet suggested that MPs may act as scaffolds for gallstone formation, facilitating the aggregation of cholesterol and bilirubin [73]. However, as current evidence remains limited, the hypothesis that MPs could act as nucleation surfaces or structural scaffolds for gallstone formation should be considered speculative and requires further experimental validation. The formation of large lipid–microplastic hetero-aggregates is proposed to induce lithogenesis [74]. This phenomenon has been further associated also with gut dysbiosis, characterized by a reduction in beneficial bacteria (e.g., *Firmicutes*) and an increase in potentially opportunistic genera (*Fusobacteriota*, *Chlamydiae*, and *Myxococcota*) [75].

**Figure 1:**
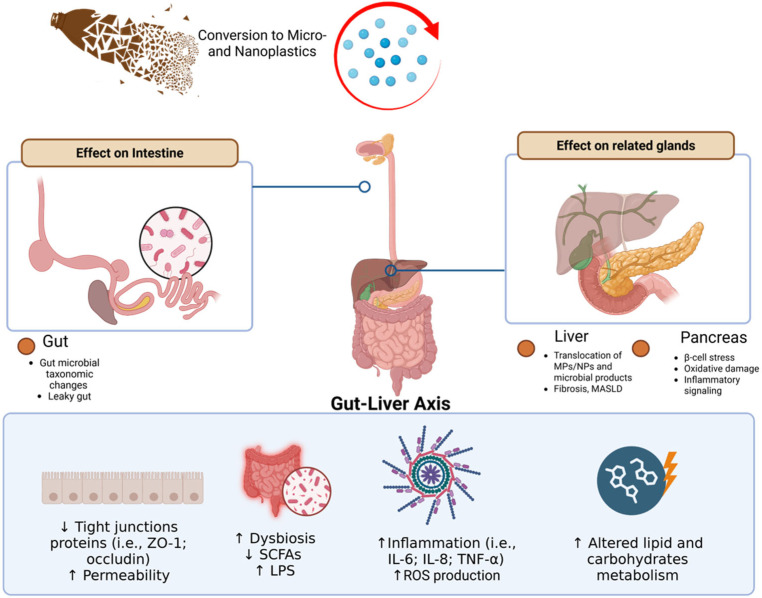
Schematic overview of the effects of MPs/NPs plastics on the digestive system and related glands. The intestine represents the primary interface of exposure, where chronic interaction with MPs/NPs perturbs epithelial architecture, tight junction organization, and gut microbiota composition, leading to functional impairment of the intestinal barrier and disruption of luminal metabolic balance. These alterations reshape the qualitative and quantitative profile of intestinal outputs entering the portal circulation, including microbial-derived components, MPs and NPs, and bioactive metabolites. At the hepatic level, this sustained exposure alters the intestinal milieu resulting in chronic metabolic and inflammatory stress, characterized by oxidative imbalance, activation of pro-inflammatory pathways, and dysregulation of lipid and carbohydrate metabolism. On the other hand, pancreatic tissues are also affected, with MPs/NPs exposure associated with oxidative stress and molecular alterations linked to β-cell dysfunction. ↑, increase; ↓, decrease. Created with BioRender.com.

An integrated conceptual framework of how MPs/NPs impact the homeostasis of the gastro-intestinal tract, and its related glands, is provided in Figure 1.

## 5. Impact of MPs/NPs on the Gut Microbiome

The gut microbiome is a complex ecosystem of bacteria, fungi, and viruses and it is indispensable for host health, playing key roles in digestion, metabolism, immune regulation, and intestinal barrier protection [76,77]. Maintaining a balanced microbiome is crucial for disease prevention and metabolic homeostasis [78]. Chronic exposure to MPs/NPs may alter microbial equilibrium, potentially promoting dysbiosis, reduced biodiversity, and shifts in the relative abundance of beneficial and opportunistic bacterial taxa, although the magnitude of these effects appears to depend on particle type, dose, and exposure duration [4,79].

From a translational perspective, while direct human clinical data on MPs-induced dysbiosis remains emerging, the risks are inferred from the observed disruption of core human-associated taxa. Murine models, which offer the closest physiological approximation to human metabolic responses, administered PS MPs/NPs over 28 days exhibited a significant reduction in microbial diversity. Specifically, smaller particles (20 μm and 0.5 μm) triggered a decrease in pro-inflammatory taxa (e.g., *Escherichia–Shigella*, *Enterobacteriaceae*), while larger particles (5 µm) showed no significant change [4]. Other rodent studies noted an increase in *Muribaculaceae* and a decrease in beneficial *Lactobacillaceae* abundance, suggesting clear compromise of the intestinal protective barrier [80]. To understand the underlying biological mechanisms, ecotoxicological models provide supporting evidence of metabolic interference. In zebrafish, exposure to PS MPs/NPs resulted in a decreased *Firmicutes*/*Bacteroidetes* (F/B) ratio and reduced *Fusobacteria*. However, the F/B ratio should not be viewed as a universal marker of dysbiosis; its significance is highly context-dependent and varies significantly across species and dietary conditions [81]. These compositional shifts in fish correlated with dysregulation of lipid and carbohydrate metabolism [81]. In *Nile tilapia*, dietary ingestion led to an increase in the phylum Firmicutes, contrasting with waterborne exposure [82,83]. In *Cyprinus carpio* (carp) and marine bivalves like *Mytilus edulis*, PS MPs promoted the expansion of pathogenic bacteria like *Shewanella* and *Plesiomonas* [82]. Finally, invertebrate models highlight the physical and enzymatic toll of ingestion. In *Drosophila melanogaster*, exposure significantly reduced the F/B ratio, while studies on *Artemia salina* (brine shrimp) showed that particles <50 µm accumulating in the digestive tract significantly reduce digestive enzyme activity, impairing nutrient absorption and fundamental metabolic pathways [78,84,85]. Together, these model organisms suggest that the primary mechanism of MPs-induced harm is a combination of physical obstruction and the subsequent alteration of microbial-driven nutrient processing.

## 6. MPs/NPs Systemic Effects: Impact of Gut Dysbiosis on Multiorgan Axes

While the gut is the primary exposure site, MPs/NPs exposure could trigger a systemic cascade by disrupting inter-organ communication axes. The communication between these distant organs is not merely structural but metabolic, and it is further reinforced by the breakdown of physical barriers.

### 6.1. The Gut–Lung Axis

Chronic oral ingestion of MPs/NPs triggers a synchronized disruption of the microbial communities in both the gastrointestinal and respiratory tracts. Recent studies in murine models, particularly those focusing on PET NPs and PS NPs, suggest the existence of a “joint dynamic” where the gut serves as the primary site of injury, with secondary effects manifesting in the lungs via the gut–lung axis [86,87]. Research indicates that sub-chronic oral exposure (typically 28 days) can lead to a significant decrease in α-diversity (richness within a single community) and shifts in β-diversity (compositional differences between communities) in both the gut and the lung [86,87,88]. In the colon, this is often marked by a specific decrease in *Lactobacillus* and an increase in Gram-negative opportunistic pathogens like *Veillonella* and *Prevotella*, while in the lung diversity loss is accompanied by a distinct increase in *Pseudomonas*, suggesting that oral ingestion may selectively promote potential pulmonary pathogens [88]. Moreover, chronic NPs ingestion has been reported to cause an imbalance in lactic acid bacteria in the gut. This may result in systemic lactate accumulation (in serum, intestine, and lung), which has been directly linked to lung tissue damage and mucosal injury [86,89]. NPs were also associated with the disruption in the production of Short-Chain Fatty Acids (SCFAs). Since SCFAs are critical for maintaining the systemic anti-inflammatory tone, their reduction in the gut may weaken the immune defenses of the lungs [5].

### 6.2. The Gut–Liver Axis

MPs/NPs-induced perturbation of the gut microbiota leads to increased intestinal permeability, or “leaky gut syndrome” in mice, due to reduced tight junction proteins and mucus depletion [90]. This hyperpermeability is critical for the activation of the gut–liver axis. It allows the influx of antigens, microbes, and their products, such as Pathogen-Associated Molecular Patterns (PAMPs)—notably LPS—into the portal circulation, which supplies the liver [91]. Mostly, LPS binding to Toll-Like Receptor 4 (TLR4) activates NF-κB, leading to overexpression of inflammatory factors (TNF-α, IL-6, and IL-1β) and contributing to hepatic inflammation and diseases like Nonalcoholic Fatty Liver Desease (NAFLD) and NAFLD-related cancer [92,93].

Recently, bio-based plastics such as PLA and polyglycolic acid (PGA) are being investigated to verify if their degradation products may be less harmful with respect to the conventional fossil-based ones such as PS and PET. In this regard and relative to the gut–liver axis, exposure to conventional MPs in zebrafish decreased tight junction proteins, facilitating LPS translocation and subsequent hepatic inflammation [94,95]. In gilthead seabream, PS MPs altered gut architecture and increased potentially harmful taxa, influencing metabolic processes related to bile acid (BA) [96]. In Nile Tilapia, PE MPs ingestion altered gut microbiota and led to altered hepatic metabolomic profiles, mainly affecting amino acid metabolism [95]. In murine models, PS MPs caused dysbiosis, leading to hepatic lipid metabolism disorder, while exposure to NPs was shown to cause gut barrier disruption and liver pyroptosis [64]. These effects were transferable via Fecal Microbiota Transplantation (FMT) from NPs-treated mice and attenuated by antibiotics, highlighting the causal role of dysbiosis [64,92]. Moreover, oral pre-consumption of PS MPs amplified the hepatotoxicity of xenobiotic substances (e.g., cyclophosphamide, a chemotherapeutic agent), suggesting MPs-induced dysbiosis increases liver susceptibility to co-stressors [97].

Similarly, the bio-based plastic MPs/NPs induced intestinal barrier disruption. For instance, exposure to PGA MPs in zebrafish disrupted the intestinal barrier, increased LPS and the neurotransmitter 5-HT (serotonin), and consequently led to hepatic disorders, lipid metabolism alterations, and neurobehavioral issues via the gut–liver–brain axis [98].

Finally, PLA MPs in mice altered gut microbiota and liver/jejunum metabolism. Airborne PLA MPs also altered lung/nasal microbiota and liver transcriptomics, suggesting an “airway microbiota–lung–liver axis” [99].

The impact of MPs/NPs exposure on the gut–liver axis is further amplified within the context of Western-style, high-fat diets (HFDs), creating a self-perpetuating cycle of dysbiosis and metabolic derangement. PS MPs have been shown to aggravate HFD-induced insulin resistance in mice and increase Gram-negative taxa (*Enterobacteriaceae*, *Prevotellaceae*), alongside elevated circulating levels of LPS and pro-inflammatory cytokines (TNF-α and IL-1β) [100]. Further evidence from mice exposed to PS microspheres demonstrated that HFD-fed animals exhibited greater weight gain, enhanced insulin resistance, and increased hepatic adiposity compared to mice receiving a regular diet. This phenotype was associated with an increased relative abundance of some Clostridia, such as *Lachnospiraceae* and *Oscillospiraceae*, and lower abundance of beneficial bacteria, including *Bifidobacterium* and *Parabacterioides.* Notably, FMT from donors co-exposed to PS and HFD led to similar metabolic disturbances in germ-free mice, establishing a causal role for MPs-induced dysbiosis [101].

Furthermore, MPs/NPs-driven dysbiosis significantly impairs microbial metabolic output, particularly the production of Short Chain Fatty Acids (SCFAs) (butyrate, propionate, and acetate)—essential mediators of barrier integrity and metabolic homeostasis [102,103]. Exposure to PS-NPs has been shown to significantly reduce colonic SCFA levels, especially butyrate, weakening the mucosal barrier and enhancing susceptibility to inflammation [4]. SCFAs exert their biological effects primarily through G-protein-coupled receptors such as GPR41 and GPR43, as well as through epigenetic modulation via histone deacetylase inhibition [104]. In the context of liver pathology, emerging evidence suggests stage-specific alterations of SCFAs during NAFLD progression. In particular, circulating SCFAs have been shown to decrease in advanced stages such as Nonalcoholic steatohepatitis (NASH) and NAFLD-related cirrhosis, and their levels are inversely associated with inflammatory markers (e.g., TNF-α), suggesting a protective anti-inflammatory role [105]. Microbiome-based studies further show that altered fecal SCFA profiles are closely associated with NAFLD phenotypes and can discriminate disease severity, linking SCFA dysregulation to hepatic steatosis and progression [106]. Furthermore, these molecules exert protective effects against diet-induced obesity [107].

In murine models, PS MPs exposure disrupts the F/B ratio and induces widespread metabolic derangements, including reduction in nucleotide and carbohydrate intermediates, accumulation of bile acids, and elevation of 2-hydroxybutyrate (a metabolite which has been consistently associated with insulin resistance) and carnitine derivatives which fuel Trimethylamine N-oxide (TMAO) production, linked to cardiovascular risk [108].

An isotope-labeling study demonstrated that PLA MPs degradation products can be assimilated as carbon sources within the gut microbiota of mice, thereby reconfiguring microbial metabolic fluxes and ultimately leading to diminished SCFAs output [109]. Photoaged PLA-NPs also caused marked dysbiosis (depletion of beneficial bacteria like *Lactobacillus*) and epithelial damage in aquatic insects [110].

In this context, in vitro systems represent a crucial tool for simulating the interactions between MPs/NPs and the human gut microbiota, overcoming ethical constraints associated with studies in vivo. Since the late 1990s, these models have combined different cell lines to mimic complex epithelial and toxicological responses [111]. Platforms like the SIMulator of the GastroIntestinal tract (SIMGI) and SIMulator of the Human Intestinal Microbial Ecosystem (SHIME) are used to verify interference with human microbiota [111,112]. Tested MPs, such as PET, reduced bacterial diversity and promoted pro-inflammatory genera (*Escherichia*/*Shigella* and *Bilophila*), which are associated with chronic inflammatory diseases. Furthermore, MPs can be colonized by biofilms, potentially acting as reservoirs for bacterial spread and antibiotic resistance [111,113].

### 6.3. The Gut–Brain Axis

The gut is the principal target of MPs/NPs toxicity, but recent studies have demonstrated that plastic particles can enter the blood circulation and traversing the blood–brain barrier (BBB), causing neurotoxicity [114,115]. As known, MPs/NPs can penetrate tissues via transport processes, such as pinocytosis, according to their size: the larger they are, the more difficult their elimination, and the longer their persistence [116]. Ingestion of PS MPs/NPs can increase intestinal permeability, inducing mucosal damage and intestinal dysbiosis, leading to anxiety-like behavioral disorder [80,117]. Furthermore, cholesterol can enhance the absorption of MPs/NPs, whereas protein molecules hinder this process [114,118,119]. Notably, as demonstrated by Chen et al. in adult murine models, dysbiosis can contribute to central nervous disorders, such as Alzheimer’s disease, insomnia, and anxiety. Indeed, the Open Field Test (OFT) they performed in their study, on exposed mice versus controls, revealed more anxious behaviors in the first group, associated with stress disorders [80].

To support this theory, Wang et al. exposed adult mice to Low-Density PolyEthylene MPs (LDPE MPs), both oxidized (Ox LDPE MPs) and non-oxidized (N-Ox LDPE MPs) [120]. Exposure to both kinds of LDPE MPs resulted in a reduction in swimming speed, deficits in learning and memory and development of depressive behavior.

MPs/NPs exposure may also trigger neurodegeneration, as found by Liang et al., where mice treated with PS NPs showed neurodegeneration due to mitochondrial dysfunction in all brain cells, microglia inflammation, energy metabolism disorders, and impairment in synaptic regulation [121]. Supporting these observations, Liu et al. showed that anionic PS NPs can bind to α-synuclein which rapidly promotes α-synuclein fibril formation and fibril-seeded pathology in cultured mice neurons. The overall findings suggest that MPs/NPs crossing the intestinal barrier and the BBB may be implicated in Parkinson’s or Alzheimer’s pathogenesis, as they are linked to neuronal loss and aberrant amyloid aggregation [122]. However, preclinical and clinical studies on neurotoxicity and neurodegeneration of both biodegradable and conventional MPs/NPs are still scarce and further studies are needed.

## 7. MPs/NPs Biological Impact on the Liver from In Vitro Models to Epidemiological Data

In vitro exposure of HepG2 cells to 1 µm PS MPs showed reduced cellular proliferation and the downregulation of the ROS detoxification enzymes glyceraldehyde-3-phosphate dehydrogenase (GAPDH), SOD2, and CAT [123]. Paul et al. used an in vitro model of the intestinal–liver axis, combining Human Colorectal Carcinoma (Caco-2) and Human Hepatic Carcinoma (HepaRG) cells that can differentiate into functional hepatic cells to better mimic human xenobiotic metabolism. They examined the uptake and transport of three different food-related polymers: PolyLactic Acid (PLA), Melamine-Formaldehyde resin (MF), and PMMA. These MPs, subMPs and NPs are very commonly used in food packaging, and they can easily cross through the intestinal barrier. This study demonstrated that all of these particles can cross the intestinal barrier and reach hepatic cells, inducing inflammatory processes in both compartments [124]. Nevertheless, it should be noted that these models have intrinsic limitations, as immortalized cell lines do not fully recapitulate the metabolic and functional properties of primary cells. Therefore, while they provide valuable mechanistic insights, their translational relevance remains limited.

Evidence from these advanced cell culture models further corroborates that exposure to MPs in a three-dimensional (3D) model incorporating various liver cell types (Kupffer cells, endothelial cells, and stellate cells) induced dose- and time-dependent cell death due to inflammation and upregulation of IL-6, IL-8, and TNF-α [104].

A recent advance in biomedical research is represented by the employment of organoids that allow the recreation of physiologically relevant 3D tissue architectures that more closely resemble in vivo organ design. Compared with conventional two-dimensional (2D) cell cultures, organoids preserve key structural and functional characteristics of native tissues, including cellular heterogeneity, spatial organization, and cell–cell as well as cell–matrix interactions. They have been used to simulate transport and adsorption of MPs/NPs, predicting their effects on health [125]. GI organoids are often organized as 3D models derived from Adult Stem Cells (ASCs) also used to test and evaluate the toxic effects of heavy metals [126]. These models have highlighted the accumulation of PS NPs (~50 nm in size) primarily in goblet and Paneth cells, contributing to the onset of an inflammatory process that culminates in apoptosis [127]. Another study demonstrated that MPs accumulated in mouse organs, including the colon, liver and pancreas after one week of exposure and induced systemic effects such as hypersecretion of inflammatory cytokines in both the animal model and in colon organoids [128]. At the hepatic level, ferroptosis induced by PS MPs led to hepatocyte death as a final consequence of lipid peroxidation [129]. Moreover, treatment of hepatic organoids with PS MPs resulted in the upregulation of Hepatocyte Nuclear Factor 4 Alpha (HNF4A) and Cytochrome P450 2E1 (CYP2E1), which actively contributes to oxidative damage through ROS generation, alongside alterations in HNF4A-mediated metabolic pathways [130]. Finally, a Gut–Liver-On-a-Chip (GLOC)-based study showed that increased intestinal peristalsis was inversely correlated with oxidative stress levels in hepatocytes, the primary MPs storage site, suggesting a complex interplay between the two organs [131].

Although these 3D models have marked a significant advancement compared to conventional animal or in vitro models—offering a multi-organ perspective that is more complex and integrated—they still present important limitations, such as the absence of components capable of mimicking vascularization or monitoring chronic and systemic effects over time [132,133].

In line with these observations, C57BL/6 mice administered with 80 nm-sized PS NPs for 28 days were analyzed for liver function [134]. The results showed increased levels of Alanine Transaminase (ALT), Aspartate Transaminase (AST), Alkaline Phosphatase (ALP), malondialdehyde (MDA) and ROS associated with enhancement of adenosine triphosphate (ATP), SOD, CAT, and GPx activities. Furthermore, PS NPs exposure resulted in considerably lower mRNA expression of Hmox1, Sod3, and RORγ in the liver, indicating that PS NPs can cause epigenetic alterations [134]. In the framework of gut–liver crosstalk, 21-day exposure of mice to PS NPs demonstrated that this treatment activated inflammatory NF-κB/NLRP3 pathways and induced expression of the cytokines IL-1β and IL-18 in the gut, which in turn recruited innate immune cells. Concurrently, a significant decrease in the expression levels of intestinal tight junction proteins (Claudin-1, Occludin, and zonulin, ZO-1) was observed, resulting in an increase in intestinal permeability and elevated LPS endotoxin levels. The high levels of LPS further activated Toll-like Receptor 4/Nuclear factor-κB/Gasdermin (TLR4/NF-κB/NLRP3/GSDMD) pathways in the liver, inducing liver inflammation and hepatocyte pyroptosis [135].

Finally, the establishment of a causal relationship between MPs/NPs exposure and the incidence of hepatobiliary carcinogenesis is hindered by the lack of environmental biomonitoring, methodological heterogeneity, difficulty in reconstructing cumulative exposure, and the long latency periods characteristic of hepatic cancers. Specific populations with chronic exposure risk (coastal residents, agricultural workers, and plastic manufacturing employees) require further investigation. The few available data from cohort studies indicated an increase in mortality from angiosarcoma of the liver (ASL) or primary HCC among 9951 men employed between 1942 and 1972 at 35 US vinyl chloride (VC) or PVC plants followed for mortality through 31 December 2013 [136]. The study indicated increased risks of ASL and HCC only in workers with high cumulative exposures—exceeding 1000 ppm-years—and after prolonged latency periods, with median latencies of 36 years for ASL and 48 years for HCC. Conversely, an 8-year extension of follow-up in 12,700 male workers in the VC industries across four European countries showed lower overall mortality than expected. However, deaths from primary liver cancer were significantly elevated. A total of 53 liver cancer deaths and 18 incident cases were identified, including ASL, HCC, and other cancer types [137]. Poisson regression analyses revealed a strong exposure–response relationship for all liver cancers, strongly suggesting that higher cumulative exposure to VC was associated with increased risk at exposure levels below 1500 ppm-years.

While increased risks of liver cancers are documented in workers exposed to VC, the carcinogenic potential of other MPs, such as PS and PE, remains poorly characterized [138]. Concerning epidemiological studies, it should be mentioned that most available studies are observational, often cross-sectional, limiting causal inference, and are frequently constrained by small sample sizes. Furthermore, preexisting conditions—especially those affecting liver health—may interact with MPs/NPs exposure, with pro-inflammatory effects and metabolic alterations potentially exacerbating underlying disease processes. Finally, the long latency of liver carcinogenesis poses an additional challenge in establishing temporal and causal relationships.

## 8. MPs/NPs-Induced Immune Molecular Pattern Activation Along the Gut–Liver Axis

**Figure 2:**
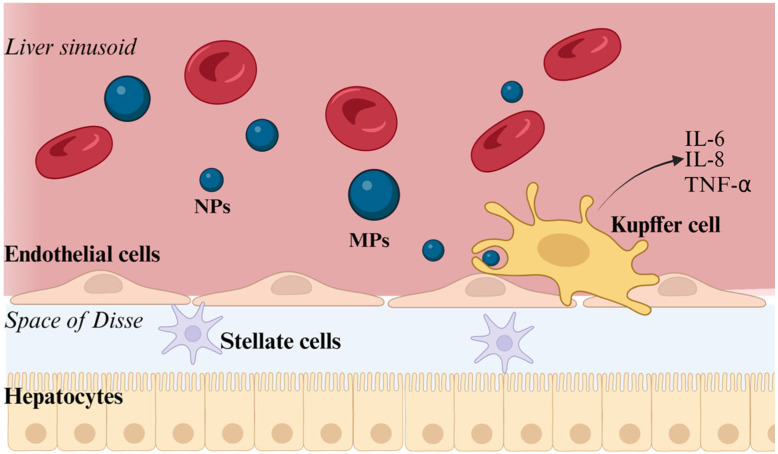
Kupffer cell-mediated response to MPs and NPs within the hepatic sinusoid. Kupffer cells are activated in the hepatic sinusoidal microenvironment following internalization of plastic particles (MPs/NPs). This process is associated with the release of pro-inflammatory cytokines, including IL-6, IL-8, and TNF-α, thereby shaping a local inflammatory response. Created with BioRender.com.

MPs/NPs are increasingly recognized as environmental stressors capable of activating immune pathways in the digestive system. Within the framework of the gut–liver axis, these particles may act as exogenous stimuli that perturb intestinal immune homeostasis and promote inflammatory signaling that propagates to the liver through the portal circulation [139]. Indeed, the GI tract represents the primary interface between MPs/NPs and the host immune system. Due to their small size, they can interact with epithelial cells and gut-associated lymphoid tissue (GALT), including Peyer’s patches, where they are internalized by antigen-sampling microfold (M) cells and processed by resident immune cells [140,141,142]. This interaction can trigger oxidative stress and inflammatory responses in intestinal macrophages and epithelial cells, contributing to the disruption of barrier integrity and increased intestinal permeability [143]. In addition, the gut-derived signals are continuously sensed by hepatic immune cells, particularly Kupffer cells, which act as a major immunological filter [144]. Once translocated across the intestinal barrier, MPs/NPs could enter blood vessels and interact with plasma proteins. This accounts for a protein corona signature influencing macrophage phagocytosis and activating specific cell death pathways. Recent findings corroborate this issue, indicating that low- and high-density polyethylene (LDPE and HDPE) NPs can be internalized via lipoprotein receptor-mediated pathways, including LDLR and SR-B1, facilitated by apolipoproteins such as ApoE. This mechanism is particularly relevant in the liver, where lipoprotein uptake is tightly regulated and central to hepatic immune function. Upon internalization, HDPE and LDPE NPs have been shown to differentially activate the caspase-3/GSDME axis, triggering a non-canonical form of pyroptosis distinct from the classical GSDMD-dependent pathway. In hepatic contexts, such activation may contribute to immune dysregulation by promoting inflammatory cell death in liver immune populations, thereby amplifying pro-inflammatory signaling and potentially impairing liver immune homeostasis (Figure 2).

A functional 3D liver microtissue model containing hepatocytes, Kupffer cells, sinusoidal endothelial cells and hepatic stellate cells has shown that PS MPs induced a dose- and time-dependent accumulation of particles in Kupffer macrophages accompanied by increased secretion of IL-6, IL-8 and TNF-α, suggesting the establishment of a pro-inflammatory microenvironment [104].

In vivo evidence further supports the involvement of innate immune pathways in plastic-induced hepatic inflammation. Oral administration of PS MPs (0.5 μm) to C57BL/6J mice for four weeks resulted in significant upregulation of pro-inflammatory cytokines including IFN-γ, TNF-α, IL-1β, IL-6 and IL-33 in liver tissues, together with infiltration of natural killer (NK) cells and macrophages into non-parenchymal compartments [145]. Other studies have reported that MPs exposure can increase intestinal permeability and promote local inflammatory mediator release without major changes in immune cell numbers, suggesting that cytokine signaling may represent early events in the gut-driven propagation of hepatic inflammation [146]. Importantly, these interactions may trigger inflammatory cell death pathways such as pyroptosis within liver macrophages. Indeed, PS MPs have been shown to induce pyroptosis in Kupffer cells via GSDMD-dependent mechanisms, leading to increased production of IL-1β, IL-6 and TNF-α both in vitro and in vivo. Genetic ablation of GSDMD or pharmacological inhibition with necrosulfonamide (NSA) significantly attenuated these inflammatory responses, highlighting the relevance of pyroptotic pathways in MPs-induced liver injury [147].

These findings underscore the importance of considering polymer composition and particle size when evaluating the immunotoxicological impact of MPs/NPs along the gut–liver axis. A synopsis of the immune and pro-inflammatory effects of MPs/NPs is given in Table 1.

## 9. Strategies for Primary Prevention and Exposure Mitigation

MPs/NPs contamination is a major global environmental concern [149]. While early mitigation efforts focused heavily on the drinking water supply chain, where surveys revealed MPs in 81% of tap water and 93% of bottled water samples, effective primary prevention requires a holistic approach addressing ingestion, inhalation, and dermal pathways across diverse environments [150,151,152].

### 9.1. Water Treatment and Point-of-Use (POU) Mitigation

Point-of-use (POU) water filtration devices have become increasingly popular among consumers [153,154]. These systems are commonly used to remove contaminants such as heavy metals (lead, arsenic, and copper), fluoride, nitrate, and compounds affecting taste and odor from drinking water. For this purpose, Cherian et al. evaluated the removal of MPs from treated drinking water by comparing the relative performance of three common POU devices and non-woven membranes (NWMs). It was demonstrated that MPs removal is most effective in POU devices equipped with the smallest pore-size membrane filter NWMs, whereas systems relying solely on Granular Activated Carbon (GAC) and Ion Exchange (IX) performed poorly without a physical barrier [155].

At the industrial level, Coagulation/Flocculation with Sedimentation (CFS) alone is largely ineffective (removal <2%), while granular filtration can achieve 86.5–99.9% removal for particles >100 µm [156]. However, particles <10 µm frequently bypass these systems, highlighting a critical gap and a need for further research on the fate of smaller MPs (<10 µm).

### 9.2. Food Processing, Packaging, and Dietary Exposure

Beyond water, the food industry represents a significant exposure vector. Primary prevention must shift toward “plastic-free” processing environments to minimize secondary contamination during mechanical shedding. The replacement of plastic-based conveyor belts, storage vats, and single-use packaging with stainless steel or bio-based alternatives is essential to reduce the “background” plastic load in processed foods [157]. Furthermore, reducing the use of heat-sealed plastic packaging for “ready-to-eat” meals is critical, as thermal stress is known to accelerate the release of MPs into the food matrix [158].

### 9.3. Occupational and Medical Exposure Mitigation

Occupational health protocols must be updated to include specialized respiratory protection (e.g., N95 or higher) and enhanced ventilation systems in industries involving textile manufacturing, plastic recycling, and 3D printing, where airborne synthetic fibers are prevalent [159]. In clinical settings, “medical-grade” exposure represents a direct route for MPs/NPs into the systemic circulation. Strategies to mitigate this include the adoption of PVC-free and Di(2-ethylhexyl)phthalate (DEHP)-free IV tubing and the implementation of inline filters for parenteral nutrition and blood transfusions to intercept micro-debris before it enters the venous system [160].

### 9.4. Regulation, Standardization, and Biomonitoring

To move from fragmented observations to a structured public health response, there is an urgent need for the standardization of biomonitoring. Current studies suffer from a lack of interlaboratory comparability. Regulatory frameworks must mandate standardized reporting units (e.g., mass concentration vs. particle count) and validated protocols for detecting the NPs/MPs in human tissues [161]. Only through such global standardization can we establish evidence-based “Tolerable Daily Intake” (TDI) limits for MPs/NPs, similar to those existing for heavy metals and persistent organic pollutants.

## 10. Conclusions

A growing body of evidence supports the role of MPs/NPs as biologically active environmental stressors with the capacity to disrupt systemic homeostasis. A central aspect of current evidence is the pivotal role of the gut as both an exposure gateway and a biological amplifier. MPs/NPs impair intestinal barrier integrity, induce oxidative stress and inflammation, and alter gut microbiota composition, promoting dysbiosis and altered intestinal permeability facilitating the translocation of microbial-derived products and plastic particles along multi-organ axes. Within this context, the gut–liver axis emerges as a central mechanistic framework linking environmental exposure to systemic disease including metabolic disease progression and potential inflammation-driven carcinogenic risk.

Immune dysregulation emerges as a unifying feature across these organs. Although the available data are still largely limited to a restricted number of non-biodegradable polymers, exposure to MPs/NPs consistently promotes a pro-inflammatory status, predominantly involving the innate immune compartment. In this context, intestinal macrophages and hepatic Kupffer cells represent key cellular targets, actively internalizing plastic particles and undergoing functional reprogramming.

In addition, the pancreas emerges as a potentially relevant—yet still insufficiently characterized—MNPs/NPs target. Current evidence is largely limited to preclinical models, where MPs/NPs have been associated with β-cell stress, inflammatory signaling, and metabolic dysregulation, suggesting a possible contribution to impaired insulin homeostasis. However, the absence of direct human data and the heterogeneity of experimental systems significantly limit the strength of these observations.

Notably, the functional interplay between pancreatic alterations and hepatic metabolism remains largely unexplored, despite the central role of insulin in coordinating liver glucose and lipid homeostasis. Furthermore, the possibility of bidirectional trafficking of MPs/NPs between the intestine, liver, and pancreas has not been systematically investigated and may represent an additional layer of complexity in exposure dynamics.

Importantly, MPs/NPs-induced effects should be interpreted within a “multiple-hit” framework, in which environmental exposure to MPs/NPs acts in concert with dietary, metabolic, and chemical co-stressors. Indeed, MPs/NPs toxicity cannot be attributed exclusively to polymer particles, as plastic-associated additives and plasticizers—such as BPA and phthalates—can leach from MPs/NPs and independently or synergistically contribute to inflammatory and metabolic disturbances.

An additional and largely unresolved issue concerns the toxicological relevance of plastic degradation products. Environmental aging processes generate complex mixtures of transformation by-products, including Non-intentionally Added Substances (NIAS), whose biological effects remain poorly characterized. The limited understanding of these heterogeneous compounds represents a critical gap in the current literature and underscores the need to integrate environmentally aged (bio)plastics into biosafety and toxicological assessments, rather than relying solely on pristine materials.

Moreover, significant limitations still hinder the translation of current knowledge into clinical relevance. The lack of standardized methodologies for MPs/NPs detection and quantification, the scarcity of longitudinal human data, and the absence of validated biomarkers of exposure and tissue damage represent major challenges. In addition, the heterogeneity of experimental models and the difficulty in reproducing chronic low-dose exposure conditions limit causal interpretation and comparability across studies. Future research should prioritize the development of standardized analytical approaches, the identification of reliable biomarkers of exposure and effect, and the integration of multi-omics data with environmental and clinical datasets. Advanced experimental systems, including organoids and multi-organ platforms, will be essential to better reproduce chronic low-dose exposure and to bridge the gap between mechanistic evidence and human pathophysiology.

From a preventive perspective, current mitigation strategies remain only partially effective. Conventional water treatment processes show variable efficiency in removing MPs, particularly in the smaller size ranges, and point-of-use filtration systems, while promising, require proper maintenance and standardization to ensure consistent performance. These limitations highlight the need for improved technological solutions, as well as for regulatory frameworks aimed at reducing environmental plastic burden and human exposure.

Notably, toxicological evidence on environmental MPs/NPs should not be interpreted as intrinsically condemning polymer technologies, but rather as providing critical mechanistic knowledge to inform risk–benefit evaluations and safe-by-design strategies. This distinction is particularly relevant when considering that polymer-based micro- and nanomaterials are also specifically engineered for drug delivery systems, where composition, degradability, and biological interactions are carefully optimized.

Advancing the understanding of the plastic–gut–liver axis, particularly under conditions of chronic exposure and real-world chemical complexity, will be essential to delineate disease-susceptibility trajectories and to inform future preventive, clinical, and regulatory approaches.

## Figures and Tables

**Table 1:** Immune and inflammatory effects of MPs/NPs in the gut and liver.

In Vitro/In Vivo Model	Type of MPs/NPs and Size	Treatment/Dose/Time Exposure	Effects	Ref.
C57BL/6J mice	PS MPs, 0.5 µm	Orally, 0.5 mg/day, 4 weeks	NK and macrophage infiltration to non-parenchymal liver cells, ↑ IFN-γ, TNF-α, IL-1β, IL-6 and IL-33 mRNA	[145]
Male ICR mice	Pristine PS MPs or UV-aged PS MPs, 4–5 µm	Intratracheally, 1 mg/day, 1 week	Gut and liver morphological damage, ↑ Nrf2 and HO-1 levels in the liver	[148]
Human intestinal organoids	Fluorescent PS NPs, 50 nm	10 μg/mL, 14 days	↑ ROS production, ↑ IL-8 release; NF-κB p65 translocation	[127]
3D human liver microtissue model	PS MPs, 1 µm	3.125–25 μg/mL up to 504 h	Increase in IL-6, and TNF-α	[104]
Mouse liver Kupffer cells and Gsdmd KO Kupffer cells	PS MPs < 10 µm	1 mg/L, 24 h	Induction of pyroptosis; ↑ of IL-6, IL-1β, and TNF-α in LPS + nigericin treated groups. ↓ inflammation upon GSDMD KO treatment	[147]
C57BL/6 and Gsdmd KO mice	PS MPs < 15 µm	Subcutaneous administration, 10 mg/kg, 20 days	Induction of pyroptosis; ↑ of IL-6, IL-1β, and TNF-α and liver injuries; Inflammatory response was mitigated in GSDMD KO mice	[147]

PS, polystyrene; Nrf2, Nuclear factor erythroid 2-related factor 2; HO-1, heme oxygenase-1; LPS, lipopolysaccharide; gsdmd KO, Gasdermin knockout; GSDMD, Gasdermin protein; ↑, increase; ↓ decrease.

## Data Availability

No new data were created or analyzed in this study. Data sharing is not applicable to this article.

## Abbreviations

The following abbreviations are used in this manuscript:

MPs/NPs	Micro- and nanoplastics
GI	Gastrointestinal tract
DDT	Dichlorodiphenyltrichloroethane
PBDEs	Polybrominated diphenyl ethers
PMMA	Poly(methyl methacrylate)
PP	Polypropylene
PVC	Polyvinyl chloride
PE	Polyethylene
PU	Polyurethane
EVA	Ethylene vinyl acetate
PA	Polyamide
PET	Polyethylene terephthalate
PAN	Polyacrylonitrile
Py-GC-MS	Pyrolysis–gas chromatography–mass spectrometry
ROS	Reactive oxygen species
PEG	Polyethylene glycol
Deff	Effective diffusion coefficient
LPS	Lipopolysaccharide
BPA	Bisphenol A
FAT2	Fatty acid transport protein 2
NAFLD	Nonalcoholic fatty liver disease
GAPDH	Glyceraldehyde-3-phosphate dehydrogenase
SOD2	Superoxidedismutase 2
CAT	Catalase
Caco-2	Human colorectal carcinoma
HepaRG	Human hepatic carcinoma
PLA	Polylactic acid
MF	Melamine-formaldehyde resin
ASCs	Adult stem cells
HNF4A	Hepatocyte nuclear factor 4 alpha
CYP2E1	Cytochrome P4502E1
GLOC	Gut–liver-on-a-chip
ALT	Alanine aminotransferase
AST	Aspartate aminotransferase
ALP	Alkaline phosphatase
MDA	Malondialdehyde
ATP	Adenosinetryphosphate
GPx	Glutathione peroxidase
ZO-1	Zonulin
ASL	Angiosarcoma of the liver
HCC	Hepatocellular carcinoma
VC	Vinyl chloride
F/B	Firmicutes/Bacteroidetes
SCFAs	Short-chain fatty acids
PAMPs	Pathogen-associated molecular patterns
TLR4	Toll-like receptor 4
PGA	Polyglycolic acid
BA	Bile acid
FMT	Fecal microbiota transplantation
HFD	High-fat diets
TMAO	Trimethylamine N-oxide
SIMGI	SIMulator of the GastroIntestinal tract
SHIME	SIMulator of the Human Intestinal Microbial Ecosystem
BBB	Blood–brain barrier
OFT	Open field test
Ox-MPs	Oxidized MPs
LDPE-MPs	Low-density polyethylene MPs
Ox-LDPE-MPs	Oxidized low-density polyethylene MPs
N-Ox-LDPE-MPs	Non-oxidized low-density polyethylene MPs
GALT	Gut-associated lymphatic tissue
LDPE-NPs	Low-density polyethylene NP
HDPE-NPs	High-density polyethylene NP
LDLR	Low-density lipoprotein receptors
GSDMD	Gasdermin protein family member
NK	Natural killer
POU	Point-of-use
NWM	Non-woven membranes
GAC	Granular activated carbon
IX	Ion exchange
CFS	Coagulation/flocculation with sedimentation
DEHP	Di(2-ethylhexyl)phthalate
NIAS	Non-intentionally added substances
